# The expanded role of the transplant pharmacist: A 10-year follow-up

**DOI:** 10.1016/j.ajt.2023.04.032

**Published:** 2023-05-04

**Authors:** Alicia Beth Lichvar, Mary Moss Chandran, Elizabeth A. Cohen, Barrett R. Crowther, Christina Teeter Doligalski, Amanda J. Condon Martinez, Lisa M.M. Potter, David J. Taber, Rita R. Alloway

**Affiliations:** 1 Center for Transplantation, University of California San Diego Health, La Jolla, California, USA; 2 Department of Pharmacy, UNC Health, Chapel Hill, North Carolina, USA; 3 Department of Transplantation, Yale New Haven Hospital, New Haven, Connecticut, USA; 4 Department of Pharmacy, University of Colorado Health, Aurora, Colorado, USA; 5 Kidneylink, U.S. Renal Care Plano, Texas, USA; 6 Department of Pharmacy, University of Chicago Medicine, Chicago, Illinois, USA; 7 Division of Transplantation, Department of Surgery, Medical University of South Carolina, Charleston, South Carolina, USA; 8 Division of Nephrology, Department of Internal Medicine, University of Cincinnati, Cincinnati, Ohio, USA

**Keywords:** clinical pharmacy, transplantation, transplant pharmacist

## Abstract

The role of the transplant pharmacist is recognized by transplant programs, governmental groups, and professional organizations as an essential part of the transplant multidisciplinary team. This role has evolved drastically over the last decade with the advent of major advances in the science of transplantation and the growth of the field, which necessitate expanded pharmacy services to meet the needs of patients. Data now exist within all realms of the phases of care for a transplant recipient regarding the utility and benefit of a solid organ transplant (SOT) pharmacist. Furthermore, governing bodies now have the opportunity to use Board Certification in Solid Organ Transplant Pharmacotherapy as a mechanism to identify and recognize specialty knowledge and expertise within the field of SOT pharmacotherapy. The purpose of this paper is to provide an overarching review of the current and future state of SOT pharmacy while also identifying major changes to the profession, forthcoming challenges, and expected areas of growth.

## 1. Introduction

The scope and governance of solid organ transplantation (SOT) as a specialty within pharmacy practice have undergone a dramatic evolution. Major advances over the last 15 years, including expanded donor pools and the long-term care of transplant recipients, have necessitated the expansion of pharmacy services to meet patient care needs. Because Alloway et al^1^ described the role of SOT pharmacists in 2011, there have been extensive changes in practice. Furthermore, the landscape of responsibilities for the SOT clinical pharmacist has evolved since it was first described in 1976.^2^ These responsibilities have expanded across all phases of transplant candidate and recipient care, advocacy and access, quality assessment and performance improvement (QAPI), and clinical research. A workforce survey characterizing positions acquired by early SOT pharmacists identified a need for further expansion into ambulatory and pediatric transplant settings to match the expansion into these areas of transplant care.^3^ Additionally, governing bodies now have the opportunity to use Board Certification in Solid Organ Transplant Pharmacotherapy (BCTXP) as a mechanism to identify and recognize specialty knowledge and expertise within the field of SOT pharmacotherapy.

Therefore, the purpose of this paper is to provide an overarching review of the current and future state of SOT pharmacy although also identifying major changes to the profession, forthcoming challenges, and expected areas of growth. Key position statements for each section are detailed in Table 1.

## 2. Demonstration of Transplant Expertise

The need to demonstrate transplant pharmacotherapy expertise emerged as the Centers for Medicare and Medicaid Services (CMS) and the United Network for Organ Sharing (UNOS) set guidance for pharmacist involvement on the SOT multidisciplinary team.^4–6^ Current CMS guidance states, “The transplant center must identify a multidisciplinary transplant team and describe the responsibilities of each member of the team. The team must be composed of individuals with the appropriate qualifications, training, and experience in the relevant areas of medicine, nursing, nutrition, social services, transplant coordination, and pharmacology.”^4^ Current UNOS bylaws state, “Each transplant program should identify at least one clinical transplant pharmacist on staff who will […] be a member of the transplant team, providing comprehensive pharmaceutical care to transplant recipients,” and “The transplant pharmacist should be a licensed pharmacist with experience in transplant pharmacotherapy.”^6^ Both require that the transplant pharmacist be adequately trained and qualified, but neither defines what qualifies one to take on the role. Determining and defending the specific qualifications required when recruiting staff is the responsibility of individual transplant centers.

The most common qualification applied for and a consistent source of trained clinical transplant pharmacists are accredited postgraduate transplant pharmacist residency training programs. Pharmacy residency programs are held to rigorous accreditation standards set by the American Society of Health System Pharmacists (ASHP) that define the goals, objectives, and competencies for specialized SOT pharmacist training. The ASHP Accreditation Standards for PGY2 Solid Organ Transplant Pharmacy Training Programs were updated in 2018 to contain significant curriculum expansions. This was shortly followed by publication of the ASHP Guidelines on Pharmacy Services in Solid Organ Transplantation.^7,8^ Representing the expansion of the SOT pharmacist role, a steady growth of specialized training programs continues. Currently, there are 51 SOT pharmacy residency programs accredited or undergoing accreditation through ASHP, compared with 17 programs in 2011.^9^ Furthermore, there are SOT pharmacy fellowships that offer a higher level of research involvement with varying degrees of clinical obligations.

Substantial growth in SOT pharmacy requires the use of board certification as a preferred mechanism to demonstrate proficiency in this specialized field of practice, and governing bodies should adopt board certification as a definition of qualification. As such, the pharmacy community sought SOT pharmacotherapy specialty designation through the Board of Pharmacy Specialties (BPS).^10^ Board certification is a rigorous process conducted by an independent entity aimed at establishing criteria for a “minimally proficient specialist” and validating the training, education, knowledge base, and experience of a practitioner.^11^ Significant progress toward and achievement of BPS recognition has occurred over the past decade.^12,13^ The BPS BCTXP program was designated a specialty in 2018, followed by the first offered examination in 2021.^10^ The examination assesses advanced knowledge and skills in the following domains: clinical skills and therapeutic management; administration and practice development; information management and education; and public health. The initial examination included 210 examinees with a 72% pass rate, resulting in over 150 BCTXP-certified pharmacists.^14^

Although postdoctoral training programs have helped train a capable workforce well prepared for SOT pharmacist specialist positions, the importance of growth management needs to be recognized. The focus of such growth should be providing SOT-related pharmacotherapy expertise for all patients, whether pre-transplant or decades after the transplant event, through the thoughtful addition of appropriately trained and proficient personnel. As transplant programs send patients back to their community referring providers, it is essential that patients always have access to SOT pharmacists. Future workforce surveys and interrogations of the profession should guide personnel expansion efforts with support from both transplant and pharmacy departments.

## 3. Clinical roles in the transplant phases of care

### 3.1. Pretransplant

The evaluation of transplant candidacy is a crucial step in determining and identifying barriers to waitlist eligibility. SOT pharmacists are an integral part of the multidisciplinary team tasked with assessing and optimizing potential recipients for transplantation. Per CMS and UNOS regulations, a pharmacist is a required member of the multidisciplinary selection committee responsible for determining transplant eligibility and listing. Comprehensive reviews outlining the steps and pharmacologic considerations for the successful evaluation of abdominal and thoracic transplant candidates have been published to guide SOT pharmacists in this role.^15–18^ A study conducted on heart and lung transplant candidates demonstrated the clinically significant interventions pharmacists make to optimize pretransplant care and readiness for transplantation.^19^ These included medication reconciliation, vaccination, anticoagulation optimization, and drug allergy considerations. Another important role of the pharmacist during the pretransplant evaluation is the assessment of medications used to treat mental health and chronic pain conditions.^20^ Assessing medication adherence during the pretransplant evaluation is vital to identifying and mitigating potential barriers (eg, financial, knowledge, cognitive, logistical, and motivational).^16^ In pediatric patients, pharmacist-led vaccination evaluations improve adherence to guidelines and boost pretransplant vaccination rates.^21^ Given this growing body of evidence demonstrating the importance of pharmacists’ involvement in the pretransplant evaluation, the ASHP Guidelines on Pharmacy Services in SOT recommend including these services during the pretransplant evaluation.^8^ Dedicated infrastructure should be created to allow for accurate listing evaluation and waitlist maintenance, ensuring transplant recipients are prepared for immunosuppression requirements posttransplant. Table 2 details the roles and responsibilities a SOT pharmacist can assume in the pretransplant phase.

Direct patient care pharmacy services provided to transplant candidates can be financially supported through the CMS cost report and the associated reimbursement for the time provided to the transplant center. Successful reporting to the CMS cost report requires the use of time studies, which are conducted by key members of the transplant team. SOT pharmacist time spent dedicated to pretransplant activities should be captured appropriately on the CMS cost report with a systematic tracking method in place to justify funding to support additional pharmacist or pharmacy technician resources when needed.^22^

### 3.2. Perioperative/transplant index admission

Perioperative management of the SOT recipient is nuanced and complex, necessitating pharmacist involvement. Thus, it is not surprising that the role of SOT pharmacists during this phase of care dates back to the mid-1970s. The inpatient responsibilities of the SOT pharmacist are well described in the published literature.^1,2^ A national survey identified the predominant activities performed by SOT pharmacists during the peri-transplant phase as medication review (95%), laboratory review (92%), medication therapy management (92%), medication allergy review (88%), inpatient hospital bedside rounds (87%), medication education (79%), documentation (71%), and coordination of discharge medications (58%).^23^ Literature demonstrates that SOT pharmacist-led activities result in the provision of comprehensive, high-value care for the transplant recipient and are associated with a positive influence on patient and allograft outcomes. A systematic review addressing the roles of pharmacists and the impact of clinical pharmacy services on the care of SOT recipients identified medication counseling, medication reconciliation, and reviewing and optimizing drug therapy as pharmacist activities that improve SOT recipient outcomes.^24^ Moreover, the work of SOT pharmacists improves medication adherence, prevents morbidity, decreases health care–related costs, and reduces medication errors.^24^

The nature of the role of the SOT pharmacist in the multidisciplinary team permits the effective use of collaborative practice CPAs with SOT physicians. These formal agreements allow pharmacists to efficiently coordinate their clinical activities, optimize medication use, and ensure adherence to transplant program protocols. Interventions made by SOT pharmacists practicing with a CPA can translate into stewardship of health care resources, as evidenced by significant cost savings. One institution estimated an annual cost savings of $373,131.20 per pharmacist with a cost-benefit ratio of 2.65.^25^ CPAs can encompass a wide scope of clinical responsibilities, ranging from pharmacist-driven therapeutic drug monitoring to prescriptive authority for managing transplant-related comorbid conditions (eg, diabetes, electrolyte abnormalities, and infection prophylaxis).

Patient education is a key service provided by the SOT pharmacist during the perioperative phase. The index hospitalization provides an ideal setting for SOT pharmacist involvement in patient and caregiver teaching. This is often followed by outpatient education provided by SOT pharmacists immediately after index admission to solidify patient understanding and comfort with new medications as they transition to the ambulatory setting. Several modalities can be employed by pharmacists to achieve robust and thorough education, including direct in-person, technology-assisted, and hybrid approaches. Utilizing pharmacy trainees during their SOTexperiential training, when available, further facilitates patient education during the transplant perioperative phase.^26–29^ The need for social distancing during the Coronavirus disease 2019 pandemic has evolved through the use of virtual care technology. These advancements are effective in leveraging resources during the initial hospitalization.^30^

In addition to medication management and education, SOT pharmacists play an integral role in discharge planning by facilitating and guiding home medication procurement. This has become increasingly challenging as mandates from health insurance plans and pharmacy benefit managers place constraints on medication sources and this creates barriers to access at discharge.^31^ Additionally, medication access and acquisition can be a time-consuming process, including prior authorizations, coverage appeals, and alternative therapy recommendations. Nevertheless, it is critically important that patients possess their transplant medications at the time of discharge. This was elucidated by a survey conducted by Potter et al,^29^ in which 93% of respondents reported that the transplant program requires medications to be onsite with the patient before hospital discharge. Furthermore, 81% of respondents reported using discharge medications during medication education and assisting the patient with preparing medication adherence tools (eg, pillbox).

SOT pharmacists are essential members of the multidisciplinary care team that provides comprehensive perioperative care. They have demonstrated a significant impact on medication safety, resource utilization, and adherence to practice standards, and are uniquely qualified to provide high-value care for transplant recipients empowered through CPAs.^25,32–35^ Areas for the utilization of CPAs in the inpatient setting are detailed in Table 2. These CPAs are often focused on transitions of care, medication reconciliation, protocol medication ordering, renal dose adjustment, and therapeutic substitutions.

The specialized and unique role of the pharmacist during the perioperative care of SOT patients is essential and should be explicitly recognized as such. Although CMS regulations require pharmacology expertise on transplant teams, the CMS Interpretive Guidelines state, “While it is desirable that each multidisciplinary team include a pharmacist member, there may be other disciplines on the team who may also be qualified to provide pharmacology services. Examples of individuals other than a pharmacist who are also qualified to provide pharmacology services on the team are a physician, advanced nurse practitioner, or physician assistant.”^5^ We strongly disagree. The skills of a transplant pharmacist are unique, essential, proven, and cannot be substituted by another discipline.

### 3.3. Outpatient posttransplant

Few areas of SOT pharmacy practice have experienced as much growth in the past decade as the ambulatory care setting.^3^ Pharmacist value in providing care to ambulatory SOT patients is evident, particularly in areas including medication safety, medication adherence assessment and improvement, and early posttransplant medication management.^32,36–39^ Consistent with previous publications, this literature demonstrates a pharmacist’s role in reducing medication errors, improving medication list accuracy, addressing medication nonadherence, and bridging the transitions of care gap from index hospital discharge to the first outpatient visit (Table 2). The positive impact of a well-integrated specialty pharmacy on financial and adherence outcomes has been established.^40^ Ambulatory SOT pharmacists are often called upon to assist with advanced medication access tasks such as insurance appeals, patient assistance program applications, and drug manufacturer coupons, in addition to the other aforementioned clinical obligations.

Long-term comorbid disease management has become a growing role for SOT pharmacists as a transplant program often becomes the “medical home” for its recipients.^25,32–34^ With cardiovascular disease (CVD) being the primary cause of death with a functioning renal allograft and a high incidence of hypertension and diabetes in all SOT recipients, CVD and CVD risk-factor management is imperative to long-term patient survival.^41^ Improvements in diabetes control, blood pressure goal attainment, and adherence to guideline-recommended therapies with compelling indications are well documented with pharmacist involvement.^34,42,43^

Several novel pharmacist-led ambulatory services have been described. Pharmacist-led mobile health (mhealth) interventions have demonstrated improvements in tacrolimus intrapatient variability, significant hospitalization cost savings, and reduced medication errors, as well as improved patient engagement in blood pressure management control.^26,44–46^ Telemedicine approaches have also been well described as avenues to increase patient access to pharmacist services.^35,47^ Pharmacist-led cytomegalovirus (CMV) stewardship programs can improve the timely initiation of CMV therapies, achieve CMV eradication, and reduce CMV-related hospitalizations and CMV drug resistance.^48–50^ Furthermore, pharmacist-led stewardship initiatives have been called for within the SOT pharmacy community, with an opportunity to focus on biosimilars, biologics, and infusion therapies.^51^

A cornerstone of many successful ambulatory SOT pharmacy practices is robust CPAs as well as reimbursement models that capture the value of pharmacist services and support the continuation and expansion of those services. CPAs improve the efficiency of care provided by SOT pharmacists whereas also allowing them to practice at the highest level of their training and licensure. A thorough integration of CPAs within health care systems has demonstrated positive clinical and cost outcomes, although billing strategies have been described that make these approaches fiscally feasible.^33,52^ CPAs in the outpatient realm can be used to extend care, provide additional targeted oversight and clinical management opportunities for SOT pharmacists, and streamline medication access. In this way, outpatient CPAs differ from their inpatient counterparts, and are delineated in Table 2. However, CPA policies may encompass both areas of practice within their language and implementation to allow flexibility that prioritizes patient care.

Several decades of research and demonstration projects have established that SOT pharmacists are distinctly poised to intervene in the long-term care of the transplant recipient, ensuring optimum medication therapy management. There are clear opportunities for transplant pharmacists to expand their practice into a variety of traditional and innovative care models, particularly within the ambulatory care setting. Participation in SOT pharmacist-run clinical initiatives (eg, medication education and compliance outreach; posttransplant diabetes mellitus clinics; posthospitalization outreach; therapy oversight of patients with opportunistic infections, such as CMV or BK viremia; and renal dose adjustment report overview) are advanced opportunities for centers to allow patients to receive focused attention to specific parts of their care. Oversight of medication access under a CPA allows SOT pharmacists to send new prescriptions and refills, and make appropriate adjustments. This can maximize the use of a CPA in the outpatient setting and create efficient workflows within the transplant multidisciplinary team.

The SOT pharmacist contributes to patient outcomes not only from a medication safety and adherence perspective but also by developing, implementing, and monitoring immunosuppressive and infectious disease protocols alongside the multidisciplinary team. Contributions to developing regimens to treat donor-derived diseases help allow for the successful expansion of the donor pool.

### 3.4. Transitions of care

The contributions of SOT pharmacists are not limited to index transplant admission and long-term follow-up through transplant clinics. There is a risk for medication-related problems anytime a patient moves between health care settings, including from one acute care setting to another, from an acute care setting to/from home, from one medical practice to another, and the transition from pediatric into adult care.^53^ SOT pharmacists accommodate divergent medication-related needs into a single regimen for each recipient, assist with scheduling to accommodate patient routines with drug-specific requirements, and navigate varying institutional and insurance formularies. For success, the devil is truly in the details, and pharmacists are attuned to getting the details right.

## 4. Clinical research and QAPI

Beyond the clinical care provided by SOT pharmacists, many are also involved in clinical research and QAPI initiatives. In a workforce survey of SOT pharmacists in 2015, of 176 respondents representing 113 transplant centers, 65% participated in clinical research and 85% participated in QAPI projects.^23^ A systematic review of transplant papers from 1975 through 2017 found 547 published manuscripts with pharmacist authors; 87% of these manuscripts had a pharmacist as the first author.^54^ Given the increasing number of SOT pharmacy residencies, more SOT pharmacists are likely to be involved in clinical research over time. Further, 1- or 2-year SOT pharmacy fellowships offer formal education and training in conducting research and securing grant funding. This goes beyond the research training provided in a pharmacy residency, and the expansion of fellowship opportunities is important to increase the SOT pharmacist’s involvement as primary investigators. It is critical that those interested in research have good mentorship and the opportunity to grow in this area, and this can be further expounded on during the residency training experience or focused fellowship training in transplant clinical research.^55,56^ These experiences prime SOT pharmacists to be integral members of multidisciplinary research endeavors.

QAPI is a requirement for transplant programs from CMS, with which transplant centers must track and demonstrate improvement in self-identified metrics and provide examples of completed QAPI initiatives. Many QAPI initiatives relate to medication use, such as infection rates and antibiotic selection, rejection rates and appropriate immunosuppression, and changes to transplant protocols, necessitating the involvement and leadership of SOT pharmacists. The ASHP Discussion Guide on the Role of the Pharmacist in Quality Improvement delineates the role of the pharmacist in this arena, and the ASHP Guidelines on Pharmacy Services in Solid Organ Transplantation further identify and expound on the role of pharmacists in QAPI initiatives.^8,57^ QAPI is a coordinated, multidisciplinary endeavor, so targeted involvement by the SOT pharmacist within this realm can maximize a transplant center’s QAPI initiatives. In addition, the unique training of the transplant pharmacist and their project management expertise make them ideal candidates for leadership positions with the transplant center QAPI program. Table 2 details the roles a pharmacist can have in this research and the QAPI domain for a transplant program.

## 5. National advocacy and contribution

Just as transplant professionals take responsibility for patient care decisions, they must also take responsibility for the health care environment in which they work and in which patients obtain care. At times, policies by outside entities (eg, regulatory bodies, third-party payers, and pharmacy corporations) may harm their ability to provide outstanding patient care. As professionals, SOT pharmacists must remain aware of this possibility, seek to understand concerns as they arise, and act where appropriate. Table 3 outlines several legislative and regulatory issues that have arisen within transplant and the steps taken to resolve or improve them.^58–65^

Defining and achieving success in these areas is not always clear. What one entity endorses is occasionally at odds with what another entity considers optimal. For example, prescription benefit managers rely on drug utilization techniques to control costs, but excessive authorization controls are responsible for serious harm when medical care is delayed, denied, or disrupted in an attempt to increase profits.^66,67^ It is important to recruit and consider multiple perspectives before determining agendas and advocating for change.

Professional organizations offer an invaluable opportunity to connect individuals with shared interests, share information and expertise, deliberate complex issues, and raise a collective voice on policy issues beyond what could be achieved individually. SOT pharmacist networks exist within the American Society of Transplantation (ie, the Transplant Pharmacy Community of Practice), the American College of Clinical Pharmacy (ie, the Immunology/Transplantation Practice and Research Network), and the International Society for Heart and Lung Transplantation (ie, the Pharmacy and Pharmacology Council).^68–70^ Committees and workgroups founded within these SOT pharmacist networks work to advance the field of transplantation by designing impactful educational programming; screening for, responding to, and driving legislative and regulatory improvements; and taking action to improve patient access to medications. Additionally, SOT pharmacists can participate in other committees and areas of these professional organizations alongside physicians and other members of the multidisciplinary team to engage in meaningful national collaboration. This is made clear in the recently published consensus recommendations for maintenance immunosuppression, which were a SOT pharmacist-led effort endorsed by the American College of Clinical Pharmacy, the American Society of Transplantation, and the International Society for Heart and Lung Transplantation.^71,72^

Finally, professional advances in the realm of SOT pharmacy are not limited to pharmacists in the United States. Pharmacists working as specialists in organ transplantation are well described in Canada and the United Kingdom, with specialists also practicing in Singapore, Taiwan, and Saudi Arabia, among other countries.^73–77^

## 6. Future directions

The health care funding and reimbursement landscape in the United States is moving away from a fee-for-service system and toward value-based care, driven by unsustainable health care costs, improved quality, and a focus on preventative medicine. To incentivize quality care, good patient outcomes, and lower the cost of care, CMS has created value-based programs specifically for kidney care and kidney transplantation.^78^ In 2019, the US Department of Health and Human Services released the Advancing American Kidney Health Initiative, which aims to reduce the development of renal failure although simultaneously increasing access to transplants or home dialysis.^79^ This initiative laid the foundation for CMS to create the Kidney Care Choices (KCC) model and establish the framework to provide strong financial incentives for value-based kidney care.^80,81^ Although KCC is one of the most comprehensive value-based programs developed by CMS for patients with end-stage organ disease, its success may dictate the creation of programs for other organs. Because of these new financial incentives, value-based care organizations are growing and creating unique clinical settings in which pharmacists can provide direct patient care. These opportunities will undoubtedly grow as the private health insurance sector typically follows CMS’ example.

SOT pharmacists are uniquely trained to not only provide high-quality clinical care in this value-based model but also assume administrative responsibilities that drive the success of these programs. Regardless of the allograft, transplant patients across any phase of care are medically complex and require consistent and prospective medication optimization to ensure optimal outcomes. As described above, SOT pharmacists demonstrate expertise in complex medication management, clinical service creation, quality improvement, performance analytics, and population health, all of which are necessary components of assessing the success of value-based care models. Like other members of the care team, pharmacists have a strong understanding of the deficiencies in the US health care system, which can impact how patients interface with their providers and ultimately lead to poor outcomes. Equitable and universal access to appropriate therapeutics and monitoring are key barriers to achieving good outcomes. Specialist pharmacist education, training, and expertise in addressing these care gaps can significantly impact how value-based care programs structure workflows to address health care access issues, social determinants of health, and equitable access to care. Ensuring SOT pharmacists are included in the development of these models can only enhance the overall goals of the Advancing American Kidney Health Initiative and all future value-based care initiatives.

To support the current and future role of SOT pharmacists in the provision of direct patient care, value-based payment models should consider pharmacists as essential providers. Furthermore, in traditional fee-for-service models, pharmacists should be able to bill for direct patient care and comprehensive medication management, independent of dispensing or specialty pharmacy services. These allowances will ensure that SOT pharmacists remain essential members of the care team and that every transplant patient will have direct access to SOT pharmacy services throughout their life.

In addition to these direct patient care roles, the skills of the SOT pharmacist extend to other aspects of medicine. There has been a notable uptrend in the expansion of pharmacists in various fields outside the patient care arena. This expansion is noticed within the pharmaceutical, biomarker, contract research, and pharmacy benefit manager industries, in addition to other roles within the transplant program such as administrative leadership. How these new nonpatient-facing roles will evolve has yet to be fully realized. However, the specialty training and skillset in project management make this area ripe with an opportunity for SOT pharmacist contribution.

## 7. Conclusion

Over the last decade, SOT pharmacist activities have expanded in both patient-facing and nonpatient-facing roles in service to the growing transplant patient population and the divergent settings where they receive care. Advocacy within the transplant community to identify SOT pharmacists as essential providers could facilitate this expansion by allowing a mechanism for billing services within the current health care landscape. Key position statements related to transplant pharmacy practice can be found in the Supplemental Table. The clinical benefit and cost-effectiveness of SOT pharmacist efforts are well established. Competency models are now in place to support these changes. Expansion into nonpatient-facing roles reflect the SOT pharmacists’ global knowledge base, which is crucial to transplant advancement activities in the future.

## Supplementary Material

supplemental material

## Figures and Tables

**Table 1:** Key position statements for the role and responsibilities of a solid organ transplant pharmacist.

Section	Key position statement

**2. Demonstration of transplant expertise**	• The inclusion of a clinical transplant pharmacist as a specified member of the transplant multidisciplinary team should be incorporated into the CMS guidance with specific definitions for who qualifies included both in the CMS guidance and UNOS bylaws • Substantial growth in transplant pharmacy requires the use of board certification as a preferred mechanism to demonstrate proficiency in this specialized field of practice
**3.1. Clinical role in the transplant phases of care: pretransplant**	• Pharmacist evaluations in the pretransplant phase optimize the assessment of transplant candidates and improve posttransplant education and care • Pretransplant pharmacist involvement is an important component of the CMS cost report and an ideal mechanism to support the pharmacist full-time equivalent expansion
**3.2. Clinical role in the transplant phases of care: perioperative/transplant index admission**	• Solid organ transplant pharmacists are essential members of the multidisciplinary care team that provide comprehensive peritransplant care. They have demonstrated significant impacts on medication safety, resource utilization, and adherence to practice standards and are uniquely qualified to provide high-value care for transplant recipients empowered through collaborative practice agreements
**3.3. Clinical role in the transplant phases of care: outpatient posttransplant**	• There are distinct opportunities for transplant pharmacists to expand practices into a variety of traditional and innovative care models, particularly within the ambulatory care setting • Solid organ transplant pharmacists are distinctively poised to ensure optimum medication therapy management in the ambulatory care arena for transplant recipients through carefully crafted collaborative practice agreements that maximize their scope of practice as essential providers
**4. Clinical research and QAPI**	• Solid organ transplant pharmacists are well-positioned to participate in and lead multidisciplinary QAPI and clinical research endeavors for a transplant program
**5. National advocacy and contribution**	• Transplant pharmacists are strong stewards of policies relating to medication management and access although ensuring the recognition of the role of pharmacists on transplant care teams
**6. Future directions**	• In value-based or accountable care-based payment models, pharmacists should be considered essential providers • In fee-for-service-based payment models, pharmacists should be able to bill for direct patient care and comprehensive medication management, independent of dispensing or specialty pharmacy services, to sustain and grow high-value services

QAPI, quality assessment performance improvement.

**Table 2:** Comprehensive responsibilities of the sot pharmacist within the context of a transplant program.

Pretransplant	Peritransplant	Posttransplant/outpatient	QAPI/research
- Identify contraindications to transplantation - Plan induction and maintenance of IMS regimens - Identify patients who are colonized with resistant organisms - Recommend appropriate perioperative antimicrobials based on colonization with resistant organisms - Participate in transplant candidate selection by communicating medication-related concerns - Design vaccination schedules - Assess medication adherence and health literacy level - Identify and educate low health literacy patients - Provide education on posttransplant information and expectations to all patients - Identify patients at risk of poor adherence to help overcome barriers - Identify financial or other barriers to obtaining prospective posttransplant medications - Identify any major drug interactions with IMS to potentially eliminate before transplantation	- Attend daily rounds with a prospective evaluation of individual pharmacotherapy - Coordinate development and implementation of drug therapy protocols, assist in ensuring protocol adherence, and measure outcomes with these protocols - Provide medication reconciliation, medication therapy management, and discharge counseling ^a^ - Provide pretransplant and posttransplant medication education - Optimize pharmacotherapy to maximize patient and center-specific outcomes - Provide comprehensive medication management including but not limited to therapeutic drug monitoring ^a^ - Provide pharmacotherapeutic support evidenced by daily documentation of activities in the patient’s medical record - Facilitate transplant discharge medication access by identifying and mitigating barriers to acquisition and uses - Coordinate and support effective transitions of care (between phases of transplant care, during pediatric to adult transition of care, and with transfer to other providers or institutions)	- Manage IMS regimen^a^ - Manage chronic disease states ^a^ Follow-up vaccination schedules - Ensure continued proper access to medications and medical supplies ^a^ - Practice stewardship endeavors to optimize outcomes ^a^ - Coordinate and support effective transitions of care (between phases of transplant care, during pediatric to adult transition of care, and with transfer to other providers or institutions) - Assess and encourage medication adherence by identifying and removing barriers where possible - Provide ongoing education regarding the medication regimen	- Manage projects related to transplant research and/or QAPI endeavors - Perform statistical analysis or analysis interpretation of data within the context of ongoing projects - Engage in scientific writing endeavors alongside a multidisciplinary transplant team - Submit abstracts to national conferences and manuscripts for publication - Provide education and training to members of the transplant team and practitioners in training - Composing and updating center-specific transplant medication use guidelines and protocols - Optimize electronic health records including transplant medication orderable - Assist in establishing workflows/processes to optimize transitions of care - Maintain and update order sets and standardized medication education tools - Establish a process to adhere to transplant regulations, documentation standards/templates, and center policy and procedures for multidisciplinary care

IMS, immunosuppression; QAPI, quality assurance performance improvement.

a Items that can be addressed or encompassed within a collaborative practice agreement.

**Table 3:** Transplant medication/transplant pharmacy issues requiring advocacy and action.

Entity	Problem	Solution

CMS: Termination of Medicare eligibility at 3 years post kidney transplant	For patients enrolled in Medicare through the end-stage kidney disease benefit, Medicare coverage will end 36 months after a kidney transplant.^58^ Unless they carry alternate insurance at that time, they become uninsured and are at a high risk of medication nonadherence and allograft loss	Comprehensive immunosuppressive drug coverage for kidney transplant patients was adopted via the Consolidated Appropriations Act of 2021.^63^ This new Medicare benefit offers life-long immunosuppressive drug coverage to eligible kidney transplant recipients through a new benefit termed “Part B-ID,” which will go into effect on January 1, 2023.^82^
CMS: Medicare Part B program	In May 2018, a Medicare Administrative Contractor published an article stating that immunosuppressive drugs paid for by Part B must be shipped to patients’ homes and cannot be delivered to a hospital^59^	National kidney transplant program practices regarding medication procurement and education after transplantation are summarized. This work reinforced the importance of immunosuppressive drug delivery to hospitals to facilitate a safe transition home after organ transplantation^29^ In December 2018, CMS issued a clarification that immunosuppressive drugs may indeed be delivered to a transplant facility up to 2 days before discharge^64^
CMS: Medicare Part D program	The Medicare Payment Advisory Commission (MedPAC) has repeatedly advised removing immunosuppressant drugs as a protected class. Removal as a protected class could limit which drugs are on a plan formulary as well as allow plans to implement prior authorization or step therapy requirements intended to steer beneficiaries to preferred alternatives^60^	Letters of opposition have been successful to date in maintaining immunosuppressant medications as a protected class^65^
CMS: Medicare Part D program	Medicare Part D plans may use CMS-approved compendia for the determination of appropriate off-label medication indications. However, out-of-date guidance in the compendia led to inappropriate denials of claims for immunosuppressive drugs^61,62^	Letters to the 2 CMS-approved compendia have prompted action by Micromedex.^83–85^ Several additional off-label uses are now endorsed by Micromedex, with improvements ongoing.^86^
CMS: Interpretive guidelines	The current interpretive guidelines suggest that individuals such as physicians or advanced practice providers can serve as pharmacology experts on a multidisciplinary transplant team.^5^ This is a dangerous presumption that fails to acknowledge the unique training, competence, and service offered by pharmacists	Not yet solved
UNOS: Transplant program bylaws	Details regarding transplant pharmacist roles and responsibilities were stricken in a 2013 administrative rewrite	Not yet solved
Commercial insurance plans	Problematic processes include “step therapy” (eg, *Aspergillus* effective azoles in the setting of opportunistic infection prophylaxis), restricted dispensing of certain medications to mail orders or specialty pharmacies, and obtrusive processes for obtaining overrides for local fills	Not yet solved
Pharmacy corporations	Problematic policies include restricted dispensing of certain medications to specialty mail-order pharmacies with no option for urgent local fills	Not yet solved

CMS, Centers for Medicare and Medicaid Services; UNOS, United Network of Organ Sharing.

## Abbreviations:

ASHP: American Society of Health System Pharmacists
BCTXP: Board Certification in Solid Organ Transplant Pharmacotherapy
BPS: Board of Pharmacy Specialties
CMS: Centers for Medicare & Medicaid Services
CMV: cytomegalovirus
CPA: collaborative practice agreement
CVD: cardiovascular disease
HHS: US Department of Health and Human Services
IMS: immunosuppression
KCC: Kidney Care Choices
MedPAC: Medicare Payment Advisory Commission
QAPI: quality assessment, and performance improvement
SOT: solid organ transplant
UNOS: United Network for Organ Sharing
VCA: vascularized composite allografts
