# Supplementary material for: The expanded role of the transplant pharmacist: A 10-year follow-up
Source: Am J Transplant. Author manuscript; Available in PMC 2025 Feb 25. (PMC11851232; doi:10.1016/j.ajt.2023.04.032)
Supplement: supplemental material [file NIHMS2054824-supplement-supplemental_material.docx]

**Supplemental Table 1. Key Position Statements Related to Transplant Pharmacy Practice**

| **Key Position Statements** |
| --- |
| - Substantial growth in transplant pharmacy requires the use of board certification as a preferred mechanism to demonstrate proficiency in this specialized field of practice. - Pre-transplant pharmacist evaluation optimizes the assessment of transplant candidates and improves post-transplant education and care. - Pre-transplant pharmacist involvement is an important component of the CMS cost report and an ideal mechanism to support pharmacist FTE expansion. - Transplant pharmacists are essential members of the multidisciplinary care team that provide comprehensive peri-transplant care. - Transplant pharmacists have demonstrated significant impacts on medication safety, resource utilization, and adherence with practice standards. - Transplant pharmacists are uniquely qualified to provide high value care for transplant recipients empowered through collaborative practice agreements. - Transplant pharmacists are distinctly poised to intervene in the long-term care of the transplant recipient, ensuring optimum medication therapy management. - There are distinct opportunities for transplant pharmacists to expand practices into a variety of traditional and innovative care models, particularly within the ambulatory care setting. - Transplant pharmacists are well-positioned to participate in and lead QAPI and clinical research endeavors for a transplant program. - Transplant pharmacists are strong stewards of policies relating to medication management and access, while ensuring recognition of the role of pharmacists on transplant care teams. - In value-based or accountable care-based payment models, pharmacists should be considered essential providers. - In fee for service-based payment models, pharmacists should be able to bill for direct patient care and comprehensive medication management, independent of dispensing or specialty pharmacy services, in order to sustain and grow high-value services. |

CMS = Centers for Medicare and Medicaid Services; FTE = full-time employee; QAPI = Quality assurance process improvement
